# Whole-brain turbulent dynamics predict responsiveness to pharmacological treatment in major depressive disorder

**DOI:** 10.1038/s41380-024-02690-7

**Published:** 2024-09-10

**Authors:** Anira Escrichs, Yonatan Sanz Perl, Patrick M. Fisher, Noelia Martínez-Molina, Elvira G-Guzman, Vibe G. Frokjaer, Morten L. Kringelbach, Gitte M. Knudsen, Gustavo Deco

**Affiliations:** 1 Computational Neuroscience Group, Center for Brain and Cognition, Department of Information and Communication Technologies, Universitat Pompeu Fabra, Barcelona, Catalonia Spain; 2 Paris Brain Institute (ICM), Paris, France; 3 Neurobiology Research Unit, Copenhagen University Hospital Rigshospitalet, Copenhagen, Denmark; 4 Department of Drug Design and Pharmacology, University of Copenhagen, Copenhagen, Denmark; 5 Department of Clinical Medicine, Faculty of Health and Medicine Sciences, University of Copenhagen, Copenhagen, Denmark; 6 Mental Health Services, Capital Region of Denmark, Copenhagen, Denmark; 7 Department of Psychiatry, University of Oxford, Oxford, UK; 8 Center for Music in the Brain, Department of Clinical Medicine, Aarhus University, Aarhus, Denmark; 9 Centre for Eudaimonia and Human Flourishing, University of Oxford, Oxford, OX1 2JD UK; 10 Institució Catalana de la Recerca i Estudis Avancats (ICREA), Barcelona, Catalonia Spain

**Keywords:** Neuroscience, Predictive markers

## Abstract

Depression is a multifactorial clinical syndrome with a low pharmacological treatment response rate. Therefore, identifying predictors of treatment response capable of providing the basis for future developments of individualized therapies is crucial. Here, we applied model-free and model-based measures of whole-brain turbulent dynamics in resting-state functional magnetic resonance imaging (fMRI) in healthy controls and unmedicated depressed patients. After eight weeks of treatment with selective serotonin reuptake inhibitors (SSRIs), patients were classified as responders and non-responders according to the Hamilton Depression Rating Scale 6 (HAMD6). Using the model-free approach, we found that compared to healthy controls and responder patients, non-responder patients presented disruption of the information transmission across spacetime scales. Furthermore, our results revealed that baseline turbulence level is positively correlated with beneficial pharmacological treatment outcomes. Importantly, our model-free approach enabled prediction of which patients would turn out to be non-responders. Finally, our model-based approach provides mechanistic evidence that non-responder patients are less sensitive to stimulation and, consequently, less prone to respond to treatment. Overall, we demonstrated that different levels of turbulent dynamics are suitable for predicting response to SSRIs treatment in depression.

## Introduction

Major depressive disorder (MDD) is a prevalent psychiatric disorder that constitutes one of the principal causes of disability worldwide [1]. MDD imposes substantive financial and emotional burdens on individuals, their families and society [2]. MDD is a heterogeneous syndrome involving a range of symptoms, including decreased mood, anhedonia and loss of energy but also, e.g., compromised cognition, and changes in appetite, sleep, and psychomotor activity. This heterogeneity of symptom manifestations may contribute to variable treatment sensitivity, evidenced by low treatment response rates of ~50%. Only around 30% of the patients meet remission criteria after treatment with selective serotonin reuptake inhibitors [3]. The prevalence of MDD and the limited efficacy of current first-line antidepressant treatment strategies precipitate the need to describe better neurobiological mechanisms that characterize MDD and identify biomarkers that accurately predict treatment response.

Functional magnetic resonance imaging (fMRI) studies have evaluated characteristics of brain function and connectivity in depressed individuals. A particular focus has been on the brain’s intrinsic functional connectivity at rest, which in some studies has identified network characteristics associated with MDD [4–6]. Human neuroimaging studies have reported dysfunctions and abnormal communication among multiple resting-state networks (RSNs) in MDD [7–11]. The default-mode network (DMN) has received perhaps the most attention, in part because the internally oriented and self-referential thought attributed to this network presents a foundation for the neural conceptualisation of rumination in depressive patients [6, 11, 12]. Other large-scale networks, such as the executive control- and salience- networks, also seem to play an important role in depression [5, 7, 9]. However, as exemplified by DMN, the directionality of alterations has been inconsistent, even between large-scale studies aggregating data across multiple sites, with reports of both hyper- and hypo-connectivity in individuals with MDD [8, 11]. These conflicting findings support the continuous development of novel connectivity measures that may inform MDD status and treatment response. Beyond canonical functional networks, recent fMRI studies have found distributed whole-brain connectivity as well as connectivity dynamics for functional imaging markers associated with MDD [13–16], reporting altered spatiotemporal structures in depressed patients compared to controls. Thus, novel methods able to measure the complex spatiotemporal dynamics are needed to reveal how brain information processing is affected in MDD and provide crucial insights into the underlying causal mechanisms of depression.

Computational neuroscience may help explain such a complex scenario by providing brain measures that can predict treatment response and by extending our knowledge of the causal mechanisms underlying whole-brain dynamics in different subgroups of patients with MDD. Recent studies have shown that the brain exhibits signatures of turbulent dynamics [17] and have also demonstrated that this novel framework can distinguish different brain states linked to health and disease [18, 19], and to assess the functional role of long-range anatomical connections [20]. Specifically, the efficient energy cascade across scales, characterizing the turbulent regime [21, 22], is a fundamental property for brain information processing, determined by the local synchronization between brain areas at different scales. Importantly, spatial scales in the brain are defined by how local the level of synchronization is, ranging from a couple of millimeters to an almost whole-brain scale, resembling different vortex sizes in the turbulence regime of fluid dynamic. The turbulence dynamics as a global emergence from complex systems can be described by non-linear coupled oscillators [23]. Accordingly, whole-brain models based on non-linear coupled oscillators have demonstrated excellent utility in fitting the spatiotemporal dynamics in empirical functional human neuroimaging data [17]. Perturbations introduced to in silico whole-brain models combined with turbulent dynamics have implicated the role of fluctuations and oscillations in brain dynamics [24] and modeled the response capacity of different brain states to external stimulation [19, 25, 26]. This approach could help to design novel therapeutic targets for brain stimulation [27] and constitutes a potential methodological strategy for increasing our mechanistic understanding of brain information processing in MDD and possibly identifying informative predictors of antidepressant treatment response.

Here, we applied the turbulent dynamics framework consisting of complementary model-free and model-based measures to resting-state fMRI data acquired in 76 unmedicated patients with moderate-to-severe MDD and in 123 healthy controls. The patients completed an eight-week pharmacological treatment with the selective serotonin reuptake inhibitor (SSRI) escitalopram and an option to shift to second-line duloxetine at week 4 in an open-label, non-randomized clinical trial. We used the model-free approach to estimate information transmission flow across space-time scales in healthy controls and depressed patients at baseline and evaluated baseline brain measures as predictive features of antidepressant treatment response and remission at eight weeks. Finally, we applied the model-based approach to evaluate brain reactivity to external perturbations in silico. We hypothesized that these complementary measures would allow us to identify group differences in whole-brain turbulent dynamics, identify a link to treatment response and differentiate brain reactivity to external perturbation between groups.

## Methods

### Participants

Data included in the current study from patients with MDD are part of NeuroPharm, an open-label clinical trial evaluating neuroimaging, cognition, and peripheral biomarkers as predictors of treatment outcome (Clinical Trials Registration: NCT02869035). Details related to study design and methods are described here and have been described in the study protocol [28]. One hundred untreated patients with MDD were recruited from a central referral center within the Mental Health Services, Capital Region of Denmark or referred directly from one of five general practitioners. Upon inclusion, patients met DSM-5 criteria and International Statistical Classification of Diseases and Related Health Problems 10 (ICD-10) criteria for unipolar depression. All patients were moderately to severely depressed at baseline, defined by a Hamilton Depression Rating Scale-17 (HAMD-17) item score >17 [29]. Clinical diagnosis was ascertained with the Mini-International Neuropsychiatric Interview [30] and confirmed by an experienced psychiatrist. All study participants started treatment with escitalopram in flexible dosages between 5 and 20 mg per day after baseline assessments and at week 4 they could be shifted to duloxetine (dosages 30–120 mg) if not responding. Individuals considered to require other forms of antidepressant treatment were referred elsewhere and at inclusion, all patients were free of any antidepressant medication. Exclusion criteria included: prior diagnosis with primary psychiatric illnesses other than depression, current depressive episode exceeding two years, more than one previous antidepressant treatment during current depressive episode, antidepressant treatment within two months prior to the study, previous non-responsiveness or contraindications to an SSRI compound, severe somatic illness, substance or alcohol use disorder, acute suicidality, pregnancy, breast-feeding, not washed out CNS drugs, history of brain injury, sensory or cognitive impairments, contraindications to PET or MRI, insufficient language skills to undergo clinical assessments and informed consent, and age outside the range of 18–65 years old. No drugs were washed out for the purpose of this study. Patients were not financially compensated for their participation.

Healthy controls included in the current study were recruited from an online volunteer database (collected under protocols: H-15004506, H-16026898, (KF)01-2006-20). These participants were recruited for specific studies centered at the Neurobiology Research Unit (NRU), Department of Neurology, Rigshospitalet, Copenhagen, DK. Data included in the current study were drawn from the Cimbi database, a central repository of diverse data structures collected as part of studies at NRU to support consistent and quality-controlled analyses [31]. We identified 123 healthy controls who completed a resting-state BOLD fMRI scan session on the same MRI scanner as patients. The healthy control group was age- and sex-matched to the patient group and met the same inclusion and exclusion criteria as those for the patients, except the healthy individuals did not have a current diagnosis or a history of mental illness. Whereas there were no group-differences in age and sex distribution, the MDD patients had slightly less years of education 11.6 ± 1.1 vs. 11.9 ± 0.5. This is expected, given that the average age in both groups was in the mid-twenties, meaning that many of the patients got a diagnosis of MDD while still at university. The controls were generally scanned at the same scanner before or in the same period as the patients, but continuous quality controls ensured that eventual scanner software updates did not interfere with the validity. Unlike patients, healthy participants received financial compensation for participation.

### Treatment protocol

Similar to participants, the antidepressant treatment protocol has been described in detail previously [28]. After inclusion, patients completed neuropsychological testing and an MRI scan prior to treatment onset. Three non-responsive patients (i.e., <25% decrease in HAMD-6 [32]) or patients with unacceptable side effects could switch to the serotonin/noradrenergic reuptake inhibitor (SNRI) duloxetine (flexible doses: 30–120 mg/day) from week 4 onward and before week 8, consistent with clinical guidelines. Antidepressant medication was provided free of charge. Non-compliant patients, defined as having taken <66% of tablets or with serum concentrations of medicine below detection limit at week 8, were included in baseline analyses but excluded from treatment response analyses.

Depression severity (HAMD-17) and side effects were assessed by a study physician or supervised research assistant during clinical follow-up sessions at 1, 2, 4, and 8 weeks after treatment onset. The HAMD-6 (a six-item subscale of HAMD-17) was selected as the primary outcome based on evidence for superior sensitivity to change in depression severity and clinimetric properties vis-à-vis the HAMD-17 [28, 32]. No additional cognition-based therapy or other psychological treatment service was provided during clinical visits.

### Magnetic resonance imaging acquisition

MRI scan data acquisition details have been described previously [33, 34]. All MR data were acquired at Rigshospitalet (Copenhagen, DK) on a Siemens (Erlangen, DE) MAGNETOM 3 T Prisma scanner with a 64-channel head/neck coil. High-resolution, whole-brain, T1-weighted MPRAGE structural scans were acquired (inversion time=900 msec, repetition time=1900 msec, echo time=2.58 msec, flip angle=9°, in-plane matrix=256 × 256 mm, in-plane Resolution=0.9 × 0.9 mm, 224 slices, slice thickness=0.9 mm). (repetition time=2000 msec, echo time=30 msec, flip angle=90°, in-plane matrix=64 × 64 mm, in-plane Resolution=3.6 × 3.6 mm, 32 slices (acquired interleaved, bottom-up), slice thickness=3.0 mm, gap=0.75 mm, total volumes=300, scan time=600 secs). A corresponding field map was acquired to unwarp spatial distortions in EPI images.

### BOLD fMRI processing and analysis

Functional MRI data were processed and analysed using Statistical Parametric Mapping 12 (https://www.fil.ion.ucl.ac.uk/spm/software/spm12/). Functional images were corrected for slice-timing, spatially realigned, corrected for spatial distortions and co-registered to the high-resolution structural image. The high-resolution structural image was normalised into Montreal Neurologic Institute (MNI) standard space and the corresponding warping map applied to the functional images. Normalised functional images (voxel size: 2 mm isotropic) were smoothed with an 8 mm full-width half-maximum (FWHM) Gaussian filter.

### Brain parcellation

We used the Schaefer parcellation with 1000 brain areas, based on estimation from a large dataset (n = 1489) [35], to extract the time series from each subject. Furthermore, we estimated the Euclidean distances from the Schaefer parcellation in MNI space.

### Probabilistic tractography analysis

We used the Human Connectome Project (HCP) database that contains diffusion spectrum and T2-weighted neuroimaging data from 32 participants as reported previously [17]. A complete description of the acquisition parameters is described in detail on the HCP website [36]. The freely available Lead-DBS software package (https://www.lead-dbs.org/) provides the pre-processing described in detail in Horn et al. [37]. In brief, the data were processed by using a q-sampling imaging algorithm implemented in DSI studio (http://dsi-studio.labsolver.org). A white-matter mask was computed by segmenting the T2-weighted images and co-registering the images to the b0 image of the diffusion data using SPM12. For each HCP participant, 200,000 fibers were sampled within the white-matter mask. Fibers were transformed into MNI space using Lead-DBS [38]. Finally, we used the standardized methods in Lead-DBS to extract the structural connectomes from the Schaefer 1000 parcellation [35].

### Overview of the turbulent dynamics framework

**Fig. 1:**
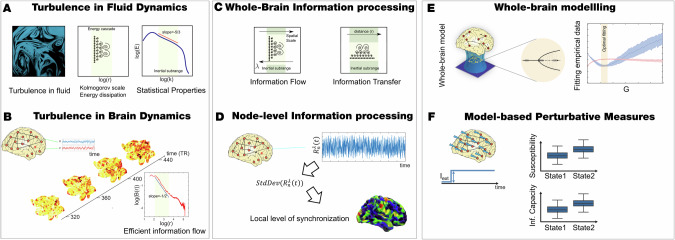
Overview of the empirical and model perturbative approaches. **A** Turbulence in fluid dynamics. Turbulence in fluids is one the most common dynamical regime where the mixing motion governs (left panel). The energy cascade, i.e., how the energy travels across scale while dissipated (middle panel) and the statistical properties defined as power laws on the energy levels and structure functions (right panel) determine the turbulent behavior of the fluid. **B** Turbulence in brain dynamics. In a recent work [17], the analysis of fMRI resting-state data in a large cohort of healthy participants (left panel) has shown the presence of turbulence as the variability of local synchronization level across time and space (middle panel). The turbulent brain regime also gives rise to the same statistical properties (right panel). **C** Whole-brain information processing. The analogy between brain activity and turbulence can also be reflected in the similarity between the local level of synchronization, determined by the local Kuramoto order parameter (R), and vortex in fluid dynamics. The turbulence regime also endows the brain with an efficient information cascade measured as the correlation of the local level of synchronization across scales (left panel). The information transfer quantified as the correlation of local synchronization across space at different scales also characterizes the brain’s information processing (right panel). **D** Node-level information processing. The node-level metastability is calculated as the standard deviation across time of the local Kuramoto order parameter. **E** Whole-brain modeling. In the Hopf whole-brain model [39, 60], the dynamics of each brain area are described through a Stuart Landau non-linear oscillator. The system of local oscillators is connected through the anatomical connectivity to simulate the global dynamics, capable of reproducing statistical observables from fMRI data (see supplementary material). We used as structural connectivity the long-range connections (LR) from human diffusion MRI measurements on top of an exponential distance rule (EDR) to fit the empirical functional connectivity as a function of the Euclidean distance (following the relation between the Kolmogorov’s second-order structure-function and the traditional FC). **F** Model-based perturbative measures. Using whole-brain modeling allows obtaining measures that rise from the perturbative approach. We simulated external stimuli and evaluated the model’s reaction for each brain state by quantifying the susceptibility and information capacity measures.

The overall methodology proposed in this work is displayed in Fig. 1 (see supplementary material for a detailed description of the framework**)**. In Fig. 1A we show that a fundamental property of turbulence is its energy cascade, i.e., the transition from large whirls to smaller whirls leading to energy dissipation (middle panel). The turbulent energy cascade presents statistical properties, as evidenced by a power law (left panel). The turbulent regime in brain activity shows the presence of highly variable, local synchronization vortices across time and space, giving rise to an efficient information cascade across scales obeying a power law (Fig. 1B). This local synchronization is determined by the local Kuramoto order parameter similar to the vortex in fluid dynamics. The scale is determined by an inverse distance parameter, *λ*, such that high *λ* values represent lower distances in the brain (e.g., $\lambda =0.01 \sim 100{mm}$ and $\lambda =0.3 \sim 3.3{mm}$). We computed the information transmission flow based on the level of local synchronization through the level of turbulence, information transfer, information cascade flow, information cascade and node-level metastability using a fine-grained parcellation of 1000 brain regions [35] (Fig. 1C, D and see more details in supplementary material). In brief, turbulence can be described as the variability in synchronization among coupled oscillators over time and space, and it is related to metastability [39]. Metastability refers to a state in which the brain exhibits a balance between stability and flexibility, allowing it to switch between different activity patterns [39], which is a global measure of synchronization. The Kuramoto local order parameter measures the synchronization level of oscillators (in this case, brain regions) over time and space, which is a local measure rather than a global as metastability. The level of turbulence is calculated as the standard deviation of the synchronization over time and space. The information cascade flow measures the transfer of information across different spatial scales over consecutive time steps by calculating the time correlation between the Kuramoto local order parameter at adjacent scales and times. The information cascade averages this flow across a range of scales to capture the overall behavior of information processing across scales. The spatial information transfer measures how information travels across space at a specific scale by computing the time correlation between the Kuramoto local order parameters of two brain areas as a function of their distance. The node-level metastability measures the standard deviation over time of the local Kuramoto order parameter (local synchronization), representing the time-averaged variance in synchronization strength of local oscillators within brain networks (for mathematical details of these turbulent-based information flow measures see supplementary material).

Complementary, the model-based approach was used to study model sensitivity to external in silico perturbations (Fig. 1E-F). For each condition, we built a whole-brain dynamical model of non-linear oscillators coupled with the DTI structural connectivity and the exponential distance rule (EDR) [17]. We assessed the reaction to the in silico stimulus with quantifiable measurements of susceptibility and information encoding capability. Susceptibility evaluates how these models react to artificial perturbations, while the information encoding capability measure captures how these perturbations are encoded within the system dynamics [19, 20].

### Support vector machine for treatment responses classification

We used a support vector machine (SVM) with Gaussian kernels as implemented in the Matlab function *fitcecoc*. The function returns a full, trained, multiclass, error-correcting output codes (ECOC) model. This is achieved using the predictors in the input with class labels. The function uses K(K – 1)/2 binary SVM models using the one-versus-one coding design, where we used K = 2 as the number of unique class labels. In other words, the SVM had seven inputs (turbulence and Information transfers at three scales and Information cascade) corresponding to the output produced by the turbulence framework analysis computed at baseline. The output was two classes corresponding to the conditions (responders and non-responders). In order to balance the classes between both labels, we considered the output from the 32 responders randomly taken from the 44 and the full output of the 32 non-responders’ patients in each repetition, subdivided into 90% training and 10% validation, repeated and shuffled 100 times. To assess the statistical significance of the accuracy/AUC values, we trained and evaluated a total of 1000 SVM classifiers using the same features (i.e., the same turbulence measurements) but randomly shuffling the class labels. We then constructed an empirical p value by counting how many times the accuracy/AUC of the classifier with scrambled class labels was greater than that of the original classifier over the 1000 SVM trained classifiers.

## Results

### Model-free framework

#### Group differences at baseline

**Fig. 2:**
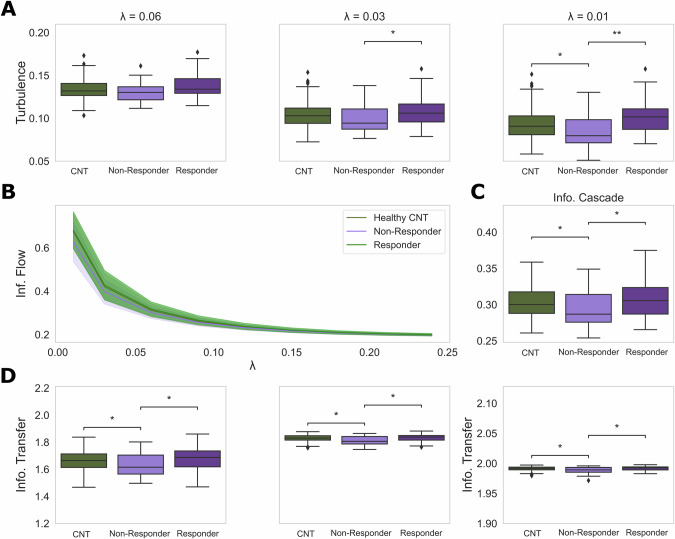
Model-free result comparison between healthy controls (CNT) and non-responder/responder patients dichotomization: Turbulence, Information transfer, Information cascade flow and Information cascade. **A** Turbulence. We computed the level of turbulence in each group to assess the spatiotemporal variability using the standard deviation of the local Kuramoto order parameter. We calculated the turbulence at different spatial scales λ = 0.01, 0.03, 0.06. The greatest differences were found at λ = 0.01, where the non-responders group showed the lowest turbulence. **B** Information cascade flow. We found that the information cascade flow significantly decreases across all scales, and for low values of λ, the non-responder group showed the lowest values. **C** Information cascade. We computed the information cascade as the average of the information cascade flow across scales indicated in panel (**B**) and we found that non-responder group showed the lowest value of information cascade compared to the other groups. **D** Information transfer. We computed the information transfer for each group and display the comparison between them for λ = 0.01, 0.03, 0.06 (left to right in figure). The non-responder group showed the lowest values of information transfer across the three λ scales compared to the other groups. P values were assessed using the Wilcoxon rank-sum permutation test (1000 permutations) and FDR corrected for multiple comparisons; ** represents p < 0.01, and * represents p < 0.05.

We compared group differences between individuals with MDD and healthy controls. We did not observe group differences in turbulence at the λ scales (Wilcoxon rank-sum permutation test, p = 0.41, p = 0.42, and p = 0.49 for λ = 0.01, λ = 0.03, and λ = 0.06, respectively). Similarly, information cascade (Wilcoxon rank-sum test, p = 0.26), and information transfer (Wilcoxon permutation test, p = 0.26, p = 0.28, and p = 0.35, for λ = 0.01, λ = 0.03, and λ = 0.06, respectively) were not significantly different between groups at baseline. Then, we dichotomized the MDD group into non-responders or responders based on their treatment outcomes, as defined in [28]. Non-responders showed lower levels of turbulence at baseline compared with responders (λ_0.06_: Cohen’s d (d) = 0.56, p_FDR = 0.07; λ_0.03_: d = 0.65, p_FDR = 0.034; λ_0.01_: d = 0.85, p_FDR = 0.006), information cascade (d = 0.59, p_FDR = 0.041), and information transfer (λ_0.06_: d = 0.51, p_FDR = 0.044; λ_0.03_: d = 0.62, p_FDR = 0.050; λ_0.01_: d = 0.67, p_FDR = 0.04). Non-responders also showed significantly lower levels of turbulence compared with healthy controls (λ_0.06_: d = 0.25, p_FDR = 0.10; λ_0.03_: d = 0.41, p_FDR = 0.069; λ_0.01_: d = 0.46, p_FDR = 0.032, information cascade (d = 0.42, p_FDR = 0.026), and information transfer (λ_0.06_: d = 0.41, p_FDR = 0.045; λ_0.03_: d = 0.47, p_FDR = 0.037; λ_0.01_: d = 0.51, p_FDR = 0.03) (Fig. 2A–D). These measures were not statistically different between responders and healthy controls. All statistical tests were false discovery rate (FDR) corrected for multiple comparison.

We present in Fig. 2 the differences in turbulence and information transfer for the three biggest spatial scales (lower values of λ) in which we found statistical significance between groups. For small spatial scales, we found no statistical differences in these measures (Supplementary Figs 1, 2). We noticed that whereas the spatial scale becomes more global (lower values of λ) the differences in the level of turbulence between non-responders and the other groups are more important and statistically significant than when measures are more local (high values of λ) (see Supplementary Fig. 3).

#### Prediction of treatment response

Next, we evaluated whether turbulence dynamics in the 76 patients with MDD as measured before treatment can predict antidepressant treatment response based on HAMD6 scores obtained before and 8 weeks after escitalopram/duloxetine treatment was been initiated. We applied a support vector machine (SVM) and 10-fold, stratified cross-validation to estimate unbiased prediction performance. Baseline turbulence dynamics features (i.e., all the turbulence-based information flow measures presented in Fig. 2) significantly predicted responder status, above chance performance (ROC-AUC: 0.70 ± 0.078, p = 0.02; accuracy: 0.63 ± 0.06, p = 0.06). Taken together, these findings support that antidepressant treatment response can be predicted above chance by whole-brain turbulence dynamics measures before the treatment is initiated.

**Fig. 3:**
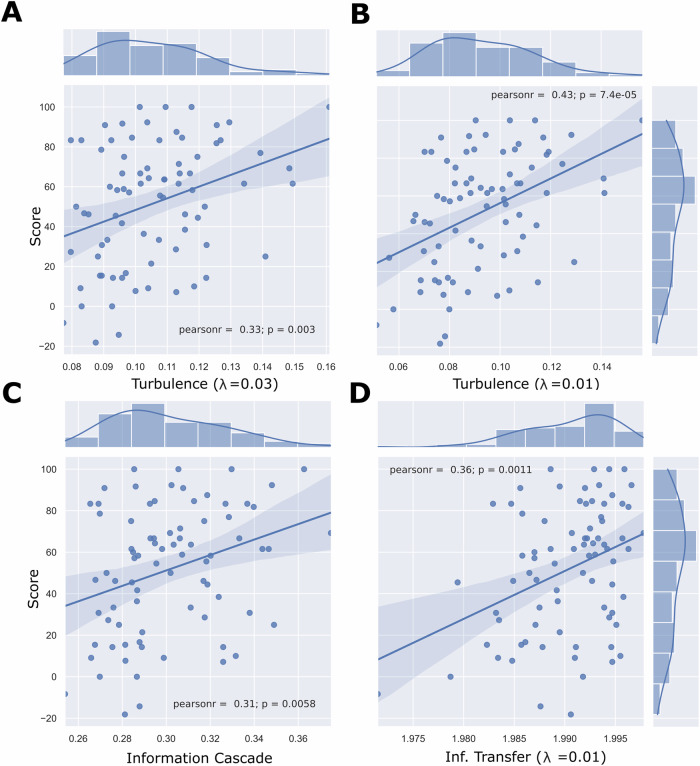
Individual level of before treatment information processing measures are positively associated with the percentage change in HAMD6 score 8 weeks after SSRI treatment has been initiated. We assessed the level of correlation between the individual levels of turbulence obtained at the baseline for patients with the percentage change in HAMD6 after 8 weeks of antidepressant treatment. **A** Correlation between the individual level of turbulence at λ = 0.03 and the percentage of change in the score. **B** Correlation between the individual level of turbulence at λ = 0.01 and the percentage of change in the score. **C** Correlation between the individual level of Information cascade. **D** Correlation between the individual level of information transfer at λ = 0.01 and the percentage of change in the score. For the chosen λ values, we find a positive and significant correlation meaning that for high values of individual patients information processing measures before treatment, the antidepressants effectiveness 8 weeks later increases.

We also investigated the relation between individual levels of turbulence, information cascade and transfer and the percentual change in HAMD6 score. We found that the percent change in HAMD6 (i.e., the more patients improved clinically) was significantly positively correlated with turbulence (λ_0.03_: Pearson’s rho (ρ) = 0.33, p = 0.003; λ_0.01_: ρ = 0.43, p < 0.001), information cascade (ρ = 0.31, p = 0.006) and information transfer (λ_0.01_: ρ = 0.36, p = 0.001) assessed at baseline in individuals with MDD (Fig. 3).

#### Node-level metastability between groups

We computed the node-level metastability across spatial scales to provide a more detailed description of information transmission at the node level. Metastability was calculated as the standard deviation over time of the local Kuramoto order parameter and indicates the variability of the level of local synchronization across time (more details in supplementary material).

**Fig. 4:**
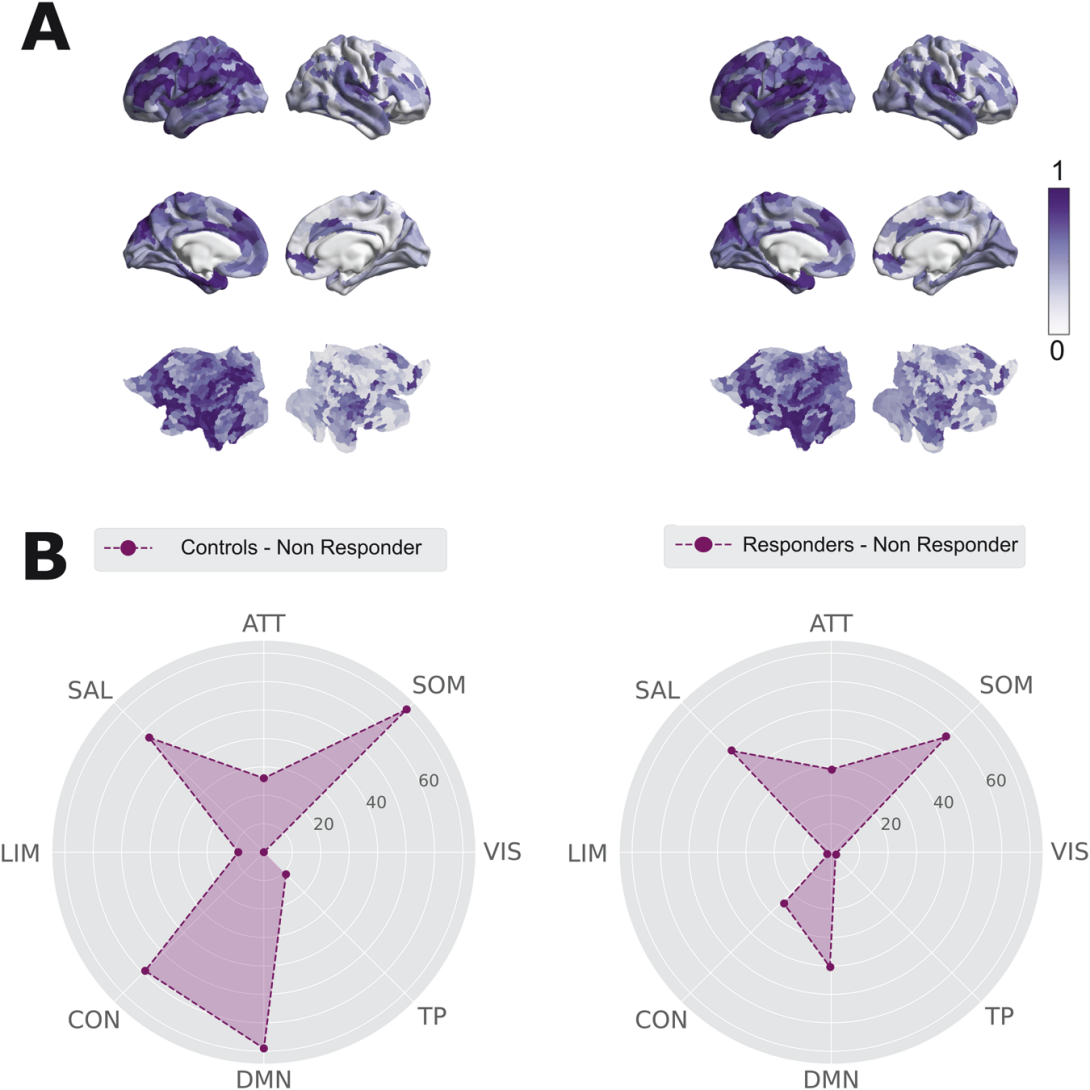
Model-free results: analysis of metastability at node-level. We computed the node-level metastability as the standard deviation across time of the local Kuramoto order parameter. **A** Brain renders represent the absolute difference of the node-level metastability between groups for λ = 0.01 normalized to the maximum value in each case. The node-level metastability was significantly lower for the non-responder group compared with controls and responder patients. **B** We selected the bottom 30% quantile of p values computed with the Wilcoxon ranksum test and FDR corrected for the 1000 node metastability differences between conditions (we display only the statistically significant comparisons). We then identified the RSNs to which they belong and quantified the number of nodes per network. Each radar plot represents the number of nodes by RSN in each comparison. The control-, salience-, attentional-, somatomotor- and default mode networks were involved in both comparisons. CON control, DMN default mode, TP temporal-parietal, VIS visual, SOM somatomotor, ATT attentional, SAL salience, LIM limbic).

We assessed differences between groups using a Wilcoxon rank-sum test with a false-discovery rate (FDR) correction across the 1000 nodes evaluated. We performed the node-by-node analysis at λ = 0.01 because our whole-brain analysis indicated this was the spatial scale most sensitive to group differences. We found that the comparisons between the non-responders vs. healthy controls, and non-responders vs. responders showed all nodes and 726 nodes with significant differences (p < 0.05, FDR corrected) in the node-level metastability, respectively. By contrast, no nodes significantly differed after FDR correction in the comparisons between healthy controls and responders. Brain renders in Fig. 4A are showing the normalized differences at node-level metastability between the non-responder group and healthy controls (first column) and between the non-responder group and responders (second column). Importantly, in both comparison the non-responder group showed lower node-level metastability values for all nodes We then selected the nodes that present a significant p value in the lower 30% quantile for each comparison and identified the RSNs to which they mainly belong. In Fig. 4B, we show radar plots of the number of nodes within each RSN that are among the 30% most significantly different.We found that the nodes of control-, salience- and default mode- networks were statistically different in non-responder patients compared to responders and healthy controls. While both comparisons (healthy controls vs. non-responders and responders vs. non-responders) showed the same RSNs differences, the differences were higher between healthy controls and non-responders in the control and default-mode networks.

### Model-based framework

We applied the model-based approach to assess how each whole-brain model reacts to external artificial stimulations by calculating the susceptibility and information encoding capability measures. We built Hopf whole-brain models of coupled dynamical oscillators using an anatomical brain architecture by linking the exponential distance rule (EDR) with the DTI connectivity matrix using a parcellation of 1000 nodes [35] (see more details in supplementary material).

**Fig. 5:**
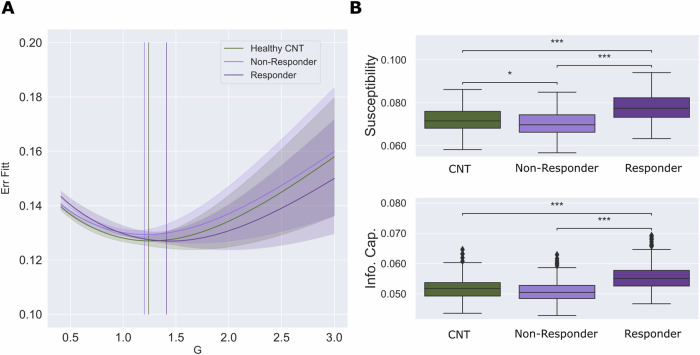
Model-based measures. **A** We present the whole-brain model FC fitting to the empirical fMRI data error as a function of the global coupling strength, G. The optimal working point of the model was defined as the minimum value of the FC fitting error, i.e., where the model shows maximal similarity to the empirical fMRI data. We found different optimal working points of the whole-brain model for each group: healthy controls (CNT), G = 1.28; non-responders, G = 1.23; responders, G = 1.55. **B** We show the results of the susceptibility (upper row) and information encoding capability (lower row) of the whole-brain models’ measures, which estimate how these models react to external artificial perturbations. The responder group showed higher susceptibility than non-responders and controls, whereas controls showed higher susceptibility than non-responders. Similarly, responders showed the highest values for the information encoding capability measure compared to controls and non-responders; however, this measure was not significant between controls and non-responders. P values were assessed using the Wilcoxon rank-sum test and corrected for multiple comparisons; *** represents p < 0.001, and * represents p < 0.05.

In particular, we fitted the functional connectivity empirical data of each group at baseline (responders, non-responders and controls) as a function of the global coupling parameter, G. This parameter measures the conductivity of fiber densities that link regions of the brain. It is determined by the underlying DTI structural connectivity, where higher values of G promote the effective transmission of information across the whole brain. The optimal working point of the whole-brain model for controls was found at G = 1.28, for non-responders at G = 1.23, and G = 1.55 for responders (Wilcoxon rank-sum test, FDR corrected: controls vs. non-responder, p_FDR < 0.001; responder vs. non-responders, p_FDR < 0.001; controls vs. responders p > 0.05) (Fig. 5A). Furthermore, we found that susceptibility was higher for responders than non-responders (d = 0.94; p_FDR < 0.001) and controls (d = 1.23; p_FDR < 0.001) and higher for controls than non-responders (d = 0.3; p_FDR < 0.05). Similarly, we found that the information encoding capability was higher in responders than controls (d = 0.82; p_FDR < 0.001) and non-responders (d = 0.81; p_FDR < 0.001). By contrast, this measure was not significantly different between controls and non-responders (d = 0.22; p_FDR = 0.1) (Fig. 5B). These results show that the responder group is not only more prone to react to the stimulation but also to encode a more complex response.

## Discussion

We applied a turbulent framework based on fluid dynamics to derive whole-brain turbulence dynamics measures and evaluate their association with MDD and their ability to predict antidepressant treatment response. We did not observe group differences at baseline between healthy controls and patients with MDD in terms of turbulence dynamics measures. Then, we dichotomized the MDD group as responders and non-responders based on their antidepressant treatment response, and found that turbulence dynamics measurers were lower for non-responder patients compared to responders and healthy controls. Importantly, higher amplitude turbulence predicts responsiveness to pharmacological treatment eight weeks after initiation and baseline turbulence measures perform significantly above chance in treatment response classification, with significant association with percent change in depressive symptom severity. Finally, using the model-based framework, we found that non-responders were less sensitive to in silico perturbations than responders and healthy controls. These results may reflect a rigidity in neural processing in non-responders that limits responsiveness to treatment. These findings provide novel evidence that turbulence dynamics at high spatial distances inform antidepressant treatment response and support whole-brain turbulence dynamics as an informative predictor of pharmacological antidepressant treatment.

### Different turbulence profiles

We investigated the turbulent profiles obtained at the baseline resting-state condition in MDD patients with different responses to the pharmacological antidepressant treatment targeting the serotonin system. We found that the level of turbulence was significantly lower in the non-responder group compared to healthy controls and responders for lower *λ* values (high distances in the brain). Specifically, the turbulence from this group was significantly smaller across three scales (*λ* = 0.06, *λ* = 0.03, and *λ* = 0.01) compared to the responder group (Fig. 2). Interestingly, we noticed that effect sizes are higher at lower *λ*, indicating that group differences in information transmission are greater at large distances. This large-scale turbulent loss indicates that the transmission of information is globally disrupted, which implicates a reduction of brain broadcast information capacity over the whole-brain network [19]. These results suggest that a reduction in the long-distance information processing could have consequences for various specialized brain processes such as action planning, verbal reporting and memory [40]. Interestingly, a decrease in turbulence at large scales is comparable with the findings presented in a previous study investigating information processing in low-level states of consciousness [18]. Importantly, our results show that the differences between the turbulence of non-responders and responders (or controls) decrease toward lower spatial scales (see Supplementary Fig. 3), similar to deep sleep [18]. These results can be interpreted as a disruption of the information processing across spacetime scales in non-responder patients. Clinically, this could imply that MDD patients with an abnormally reduced information processing capability across scales are less likely to benefit from SSRIs as first-line treatments.

### Information processing measures predict response to pharmacological SSRI treatment

We found a strong correlation between the percent change in HAMD6 score and the baseline individual levels of turbulence of all the patients **(**Fig. 3**)**. Our results revealed that higher amplitude turbulence at λ 0.01 is related to a better treatment outcome after eight weeks. However, it has been shown that these measures do not necessarily show the same tendency in all scales, as occurs with disorders of consciousness patients [19]. This finding suggests that patients with low levels of turbulence or information processing are more likely not to respond to SSRI/SNRI treatment and that, e.g., psychedelics [41] may be more efficient considered, as they have been shown to increase whole-brain turbulent dynamics [18]. We also assessed the classifier’s performance to distinguish between treatment responses. We found that the AUC of the SVM classifier trained to disentangle responders from non-responders using measures from information processing is 71%. Future investigations could combine these features from information processing measures with behavioral and cognitive assessments to improve the accuracy of the treatment response prediction.

### Node-level metastability differences between groups

We investigated the local changes in metastability using a fine-grained parcellation to identify potential group differences in brain regions potentially involved in ruling the underlying turbulent dynamics. Significantly, the analysis of the node-level metastability at the resting-state baseline condition also differentiates controls from non-responders, and non-responders from responders. In particular, the highest difference was found in the nodes belonging to the DMN, attentional-, salience-, executive control- and somatomotor- networks. These findings are in line with the triple network model [11, 42] whereby MDD is characterized by imbalance connectivity between the DMN, implied in self-referential processes, and other large-scale networks associated with cognitive control or the regulation of attention toward external stimuli such as the salience and executive control networks [7–11, 43–45]. Prior neuroimaging studies investigating the role of the DMN in MDD have reported inconsistent results. For example, some studies have found increased connectivity in the DMN [6, 11, 46], while others found reduced connectivity in MDD patients [8, 47]. Such inconsistency could be due to different reasons such as sample size, ethnic differences, or the approach used to study the DMN as, for example, selecting predefined regions of interest (ROIs), static functional connectivity or changes in the underlying dynamics.

### Causal whole-brain modeling and in silico perturbations

We explored in silico external perturbations that emulate empirical perturbation protocols. Our external perturbation operates by overall increasing the bifurcation parameter values underlying the switching of the dynamical regime of all brain region (see supplementary material). This model manipulation can be compared to a direct alteration in nodal neural excitability, which resembles the impact created by tDCS stimulation [48]. Typically, previous in silico efforts point out to assess how best to control the brain and its transitions between brain states (e.g., healthy and diseases) [49, 50]. Conversely, we wanted to investigate the causal mechanistic behind the group differences and the resulting reactivity given by external in silico perturbations, similarly to the in vivo Perturbational Complexity Index (PCI) approach proposed by Massimini and colleagues [51, 52].

This strategy proposes that the brain’s sensitivity to react to the same global external perturbations could serve as a specific biomarker revealing particular features of the dynamical complexity of brain states. Notably, the perturbative in silico approach allows the investigation of brain responses evoked by artificial stimulations, which are not limited by ethical constraints in humans [49, 53, 54]. To this end, we built Hopf whole-brain models, which link the underlying anatomy with the local dynamics of brain areas using the non-linear Stuart-Landau oscillator [55, 56]. Crucially, we found that the optimal working point of the whole-brain model for the responder group shifted to a higher global coupling value than healthy controls and the non-responder group (Fig. 5A). This drastic shift toward a higher coupling indicates supercritical behavior, which is indicative of a variation in the brain’s underlying dynamical complexity [24]. When we perturbed each whole-brain model at its optimal working point and retrieved susceptibility and information capability measures, we found an increase in these measures in the responder group compared to healthy controls. In contrast, the non-responder group showed a reduction in the susceptibility measure (Fig. 5B). A higher information capability and susceptibility, as found in the responder group, could suggest a signature of depression to characterize those patients who are more likely to benefit from SSRI treatment. In other words, a pre-existent higher information capability and susceptibility may be a prerequisite to respond to the SSRI modulation in depression [46, 57]. Nevertheless, to generate a comprehensive understanding of the relationship between information processing and treatment responses, it is important to investigate whether the turbulence measures are altered after the treatment and the mechanisms behind the dynamical changes generated by drug intervention. An interesting future direction could be to address these questions by using both model-free and model-based approaches. The model-free approach could be based on longitudinal studies, allowing the computation of empirical turbulence measures from fMRI data alongside treatment responses. Complementarily, the model-based approach could provide a mechanistic explanation for longitudinal changes in turbulence measures during patient treatment by creating and perturbing whole-brain models informed with drug-specific neurotransmitter receptor maps [58]

Importantly, this model-based approach could enlarge the horizons of possible treatments by modeling the effect of different drugs, e.g., .psilocybin [59]. Importantly, our in silico perturbation protocol operates at a global level, i.e., we randomly changed the bifurcation parameters of the whole brain simultaneously within a fixed range, obtaining global measures. However, these measures can also be computed when regional perturbations are simulated, i.e., affecting a single brain area or a small set of brain regions [24]. This approach could allow us to investigate the role of each brain region in driving changes in turbulence-based information flow measures and their crucial link to treatment response.

We want to point out some limitations of the study. Firstly, our study is a naturalistic study without a placebo arm which makes it difficult to discern eventual placebo effects. It is not unlikely that the extensive investigational program could have skewed the follow-up scan participants to responders which may be the reason why we saw a relatively good SSRI response in our cohort. Also a large proportion of our patients first depressive episodes and most likely not far progressed in disease states [28]. Whereas such a bias could alter the clinical representation and response rates, we find it unlikely that it would change the fMRI outcome measures. Secondly, this work is based on human fMRI data. Therefore, spatial and temporal scales are limited to millimeters and seconds. Furthermore, the brain parcellation utilized in this study does not include subcortical areas, which could provide valuable information for MDD patients’ assessment, particularly in limbic regions. We conducted the study using a structural connectivity (SC) template obtained as the average from a separate cohort of healthy participants from the Human Connectome Project (HCP) as reported previously [17]. It is possible that using the individual SC rather than a template, to build subject-level models could improve the results and could lead to even more specific prediction of individual treatment outcome We also want to acknowledge that to go further towards clinically implementable tools, this framework will need to be replicated in other MDD cohorts.

Overall, the results of the present work represent a major methodological advancement in search of neuroimaging biomarkers that can help us to discriminate patients with MDD for the selection of optimal treatment. Our results demonstrate an abnormally reduced turbulence level in unmedicated MDD patients who subsequently show insufficient response to SSRI treatment for 8 weeks. In addition, from causal whole-brain modeling view, our findings may contribute to a mechanistic understanding of how the turbulent dynamics relate to responsiveness to treatment in depression.

## Supplementary information

Supplemental Material

## Data Availability

The datasets contain information from a clinical population and are not publicly available due to GDPR constraints. However, the authors can provide the data upon reasonable request and will be based on a data sharing agreement.

## Code availability

All original codes to compute turbulence measures and create whole-brain models are available in https://github.com/yonisanzperl/The-Turbulent-Brain-v2. The software dependence is Matlab (2018b).
