# Supplementary material for: Whole-brain turbulent dynamics predict responsiveness to pharmacological treatment in major depressive disorder
Source: Mol Psychiatry. 2024 Sep 10;30(3):1069–79. doi: 10.1038/s41380-024-02690-7 (PMC11835742; doi:10.1038/s41380-024-02690-7)
Supplement: Supplementary file 1 — Supplemental Material [file 41380_2024_2690_MOESM1_ESM.docx]

**Supplementary Material for: “Whole-brain turbulent dynamics predict responsiveness to pharmacological treatment in major depressive disorder*”***

Anira Escrichs^1,*^, Yonatan Sanz Perl^1,2,*^, Patrick M. Fisher^3,4^, Noelia Martínez-Molina^1^, Elvira G-Guzman^1^, Vibe G. Frokjaer^3,5,6^, Morten L. Kringelbach^7,8,9^, Gitte M. Knudsen^3,5,**^ and Gustavo Deco^1,10,**^

^1^Computational Neuroscience Group, Center for Brain and Cognition, Department of Information and Communication Technologies, Universitat Pompeu Fabra, Barcelona, Catalonia, Spain.

^2^ Paris Brain Institute (ICM), Paris, France

^3^Neurobiology Research Unit, Copenhagen University Hospital Rigshospitalet, Copenhagen, Denmark

^4^Department of Drug Design and Pharmacology, University of Copenhagen, Copenhagen, Denmark

^5^Department of Clinical Medicine, Faculty of Health and Medicine Sciences, University of Copenhagen, Copenhagen, Denmark

^6^Mental Health Services, Capital Region of Denmark

^7^Department of Psychiatry, University of Oxford, Oxford, UK.

^8^Center for Music in the Brain, Department of Clinical Medicine, Aarhus University, DK.

^9^Centre for Eudaimonia and Human Flourishing, University of Oxford, Oxford OX1 2JD, United Kingdom.

^10^Institució Catalana de la Recerca i Estudis Avancats (ICREA), Barcelona, Catalonia, Spain.

***Model-free framework***

*Kuramoto Local order parameter*

The time behaviour of the modulus of the Kuramoto local order parameter for a given brain area is named as the amplitude turbulence, $R_{\lambda}\left( \bar{x},t \right)$ and is mathematically defined as:

$R_{\lambda}\left( \bar{x},t \right)e^{i\vartheta_{\lambda}\left( \bar{x},t \right)}=k\int_{-\infty}^{\infty} d\bar{x}'G_{\lambda}\left( \bar{x}-\bar{x}^{'} \right)e^{i\varphi\left( \bar{x}^{'},t \right)}$ (1)

where $G_{\lambda}$ is the local weighting kernel $G_{\lambda}\left( \bar{x} \right)=e^{-\lambda\left| \bar{x} \right|}$, $\varphi\left( \bar{x},t \right)$ are the phases of the spatiotemporal data, $k$ is the normalization factor ${[\int_{-\infty}^{\infty} d\bar{x}'G_{\lambda}(\bar{x}-\bar{x}^{'})]}^{-1}$ and $\lambda$ is the spatial scaling.

Therefore, $R_{\lambda}$ defines local levels of synchronisation at a given scale, $\lambda$, as function of space, $\bar{x}$, and time, $t$. This measure captures what call the *brain vortex space,* $R_{\lambda}$, over time, and can be related with the rotational vortices observed in fluid dynamics.

*Amplitude turbulence*

The level of amplitude turbulence, *D*, is defined as the standard deviation across time and space of the modulus of local Kuramoto order parameter (R):

$D={\langle{R_{\lambda}}^{2}\rangle}_{\bar{x},t}-{\langle R_{\lambda}\rangle}_{\bar{x},t}^{2}$ (2)

where the brackets ${\langle\rangle}_{\bar{x},t}$ denotes averages across time and space.

*Information cascade flow and Information cascade*

The information cascade flow indicates how the information travels from a given scale (*λ*) to a lower scale (*λ* − ∆*λ*, where ∆*λ* is a scale step) in consecutive time steps (*t* and *t* + ∆*t*). In this sense, the information cascade flow measures the information transfer across scales computed as the time correlation between the Kuramoto local order parameter in two consecutive scales and times:

$F\left( \lambda\right)={\langle{corr}_{t}(R_{\lambda}\left( \bar{x},t+\Delta t \right),R_{\lambda-\Delta\lambda}\left( \bar{x},t \right))\rangle}_{\bar{x}}$ (3)

where the brackets ${\langle\rangle}_{\bar{x}}$ denotes averages across time and space. Then, the information cascade is obtained by averaging the information cascade flow across scales *λ* ranging from 0.01 to 0.24, which captures the whole behaviour of the information processing across scales.

*Information transfer*

The spatial information transfer indicates how the information travels across space at a specific scale, *λ*. This measurement is computed as the slope of a linear fitting in the log-log scale of the time correlation between the Kuramoto local order parameter of two brain areas at the same scale as a function of its Euclidean distance (*r*) within the inertial subrange:

$log\left( corr_{t}\left( R_{n}^{\lambda},R_{p}^{\lambda} \right)\left( r \right) \right)=A*log\left( r \right)+B$ (4)

where *A* and *B* are the fitting parameters, and the first one, the negative slope, stands for the spatial information transfer.

*Node variability of local synchronisation: node-level metastability*

We computed the node variability of the local synchronisation as the standard deviation across time of the local Kuramoto order parameter as follows:

$NLM\left( n,\lambda\right)={\langle R_{n}^{\lambda}(t)^{2}\rangle}_{t}-{\langle R_{n}^{\lambda}(t)\rangle}_{t}^{2}$ (5)

where the brackets ${\langle\rangle}_{t}$ represent average values across time points.

Here, we used the discrete version of the node-level Kuramoto order parameter, with modulus *R* and phase *ν*, representing a spatial average of the complex phase factor of the local oscillators weighted by the coupling calculated through:

$R_{n}^{\lambda}\left( t \right)e^{i\nu_{n}\left( t \right)}=\sum_{p}^{N} \left[ \frac{C_{np}^{\lambda}}{\sum_{q}^{N} C_{nq}^{\lambda}} \right]e^{i\varphi_{p}\left( t \right)}$ (6)

where *ϕ_p_*(*t*) are the phases of the spatiotemporal data, *N* is the total amount of nodes and $C_{nq}^{\lambda}$ is the local weighting kernel between node *n* and *p,* and *λ* defines the spatial scaling:

$C_{np}=e^{-\lambda\left( r\left( n,p \right) \right)}$ (7)

where *r*(*n,q*) is the Euclidean distance between the brain areas *n* and *p* in MNI space.

To compare the node-level metastability statistics, we collected the 1000 nodes values for all participants in each group and generated the distributions. Then, we compared across states the distributions using the Kolmogorov-Smirnov distance between them. The Kolmogorov–Smirnov distance quantifies the maximal difference between the cumulative distribution functions of the two samples, where larger values stand for more significant differences between both distributions.

***Model-based framework***

*Whole-Brain Computational Model*

We constructed whole-brain dynamical models based on the normal form of a supercritical Hopf bifurcation (also known as Stuart-Landau) [39]. This type of bifurcation can change the qualitative nature of the solutions from a limit cycle that yields self-sustained oscillations towards a stable fixed point in phase space. This whole-brain computational model is characterised by a series of model parameters that rules the global dynamical behaviour. One of them is the multiplicative factor, *G*, representing the global conductivity of the fibres scaling the structural connectivity between brain areas, which is assumed to be equal across the brain [39, 40]. The other relevant parameters are the local bifurcation parameter (*a_j_*), which rules the dynamical behaviour of each area between noise-induced (*a* < 0), self-sustained oscillations (*a* > 0) or a critical behaviour between both (*a* ∼ 0) (**Fig.1C**). We optimized the model parameters to better fit the empirical functional connectivity as a function of the distance, *r*, within the inertial subrange. The models consisted of 1000 cortical brain areas from the resting-state atlas mentioned above. The underlying anatomical matrix *C_np_* was added to link the brain structure and functional dynamics and was obtained by measuring the exponential distance rule as defined in Equation (7). The local dynamics of each brain area was described by the normal form of a supercritical Hopf bifurcation, which emulates the dynamics for each brain area from noisy to oscillatory dynamics as follows:

$\frac{dx_{n}}{dt}=a_{n}x_{n}-\left[ x_{n}^{2}+y_{n}^{2} \right]x_{n}-\omega_{n}y_{n}+\nu\eta_{n}\left( t \right)$ (8)

$\frac{dy_{n}}{dt}=a_{n}y_{n}-\left[ x_{n}^{2}+y_{n}^{2} \right]y_{n}+\omega_{n}x_{n}+\nu\eta_{n}\left( t \right)$ (9)

where *η_n_*(*t*) is additive Gaussian noise with standard deviation $\nu=0.01$. This normal form has a supercritical bifurcation at *a_n_* = 0, such that for *a_n_* > 0, the system is in a stable limit cycle oscillation with frequency *f_n_* = *ω_n_*/2*π*, whereas for *a_n_* < 0, the local dynamics are in a stable point (i.e., noisy state). The frequency *ω_n_* of each brain area was estimated from the empirical fMRI data as the peak of the power spectrum.

Finally, the whole-brain dynamics was defined by the following set of coupled equations:

$\frac{dx_{n}}{dt}=a_{n}x_{n}-\left[ x_{n}^{2}+y_{n}^{2} \right]x_{n}-\omega_{n}y_{n}+G\sum_{p=1}^{N} C_{np}\left( x_{p}\left( t \right)-x_{n} \right)+\nu\eta_{n}\left( t \right)$ (10)

$\frac{dy_{n}}{dt}=a_{n}y_{n}-\left[ x_{n}^{2}+y_{n}^{2} \right]y_{n}+\omega_{n}x_{n}+G\sum_{p=1}^{N} C_{np}\left( y_{p}(t)-y_{p} \right)+\nu\eta_{n}\left( t \right)$ (11)

Where the global coupling factor G, scaled equally for each brain area, represents the input received in region *n* from every other region *p*.

*Functional Connectivity Fitting*

An adaptation of Kolmogorov’s structure-function is proposed and the variable *u* was applied to the BOLD signal of the data, instead to the velocity field of the fluid. Thus, the functional correlations between each pair of brain areas with equal Euclidean distance can be defined as:

$S(r)={\langle{(u\left( \bar{x}+r \right)-u(\bar{x}))}^{2}\rangle}_{x,t}=2[FC\left( 0 \right)-FC\left( r \right)]$ (12)

where *FC* is the spatial correlations of two points separated by a Euclidean distance r, which is given by:

$FC\left( r \right)={\langle u\left( \bar{x}+r \right)u(\bar{x})\rangle}_{\bar{x},t}$ (13)

where the symbol ${\langle\rangle}_{\bar{x},t}$ refers to the average across the spatial location $\bar{x}$ of the brain areas and time. Thus, the structure functions characterise the evolution of the functional connectivity (FC) as a function of the Euclidean distance between nodes at the same distance, which is different from the usual definition of FC that does include distance. The fitting between the empirical and simulated FC is defined as the Euclidean distance between both matrices within the inertial range as defined in Deco et al. [17].

*Susceptibility*

How the brain reacts to external stimulations is quantified by the susceptibility measure of the whole-brain model. For each coupling strength value, *G*, we generated a global perturbation by randomly changing the local bifurcation parameter, *a_n_*, in the range [-0.02: 0] for all brain regions. We repeated for each *G*, i.e., each brain condition model, 50 times the same perturbation and then we averaged across trials. Is important to notice, that perturbation is carefully defined to keep the dynamical scenario in the subcritical regime of each oscillator, and in all the cases the perturbation approaches the dynamic of the node to the critical point *a=0*. The sensitivity of the perturbations on the spatiotemporal dynamics was calculated by measuring the changes in modulus of the local Kuramoto order parameter as:

${\chi=\langle{\langle{\langle\tilde{R}_{\lambda_{s}}\left( \bar{x},t \right)\rangle}_{t}-{\langle R_{\lambda_{s}}\left( \bar{x},t \right)\rangle}_{t})\rangle}_{trials}\rangle}_{\bar{x}}$ (14)

where $\tilde{R}_{\lambda_{s}}\left( \bar{x},t \right)$ corresponds to the perturbed case, the$R_{\lambda_{s}}\left( \bar{x},t \right)$ to the unperturbed case, and ${\langle\rangle}_{t}$, ${\langle\rangle}_{trials}$ and ${\langle\rangle}_{\bar{x}}$ to the average across time, trials, and space, respectively.

*Information encoding capability*

The information encoding capability captures how the external stimulations are encoded in whole-brain dynamics. The information capability, I, was defined as the standard deviation across trials of the difference between the perturbed $\tilde{R}_{\lambda_{s}}\left( \bar{x},t \right)$ and unperturbed $R_{\lambda_{s}}\left( \bar{x},t \right)$ mean of the modulus of the local Kuramoto order parameter across time *t*, averaged across all brain areas *n* as:

${I=\langle{\langle{\langle\tilde{R}_{\lambda_{s}}\left( \bar{x},t \right)\rangle}_{t}-{\langle R_{\lambda_{s}}\left( \bar{x},t \right)\rangle}_{t})^{2}\rangle}_{trials}\rangle}_{\bar{x}}- {\langle{\langle{\langle\tilde{R}_{\lambda_{s}}\left( \bar{x},t \right)\rangle}_{t}-{\langle R_{\lambda_{s}}\left( \bar{x},t \right)\rangle}_{t}\rangle^{2}}_{trials}\rangle}_{\bar{x}}$ (15)

where the brackets ${\langle\rangle}_{t}$, ${\langle\rangle}_{trials}$ and ${\langle\rangle}_{\bar{x}}$ denote the averages defined as above.

# ***Supplementary figures***


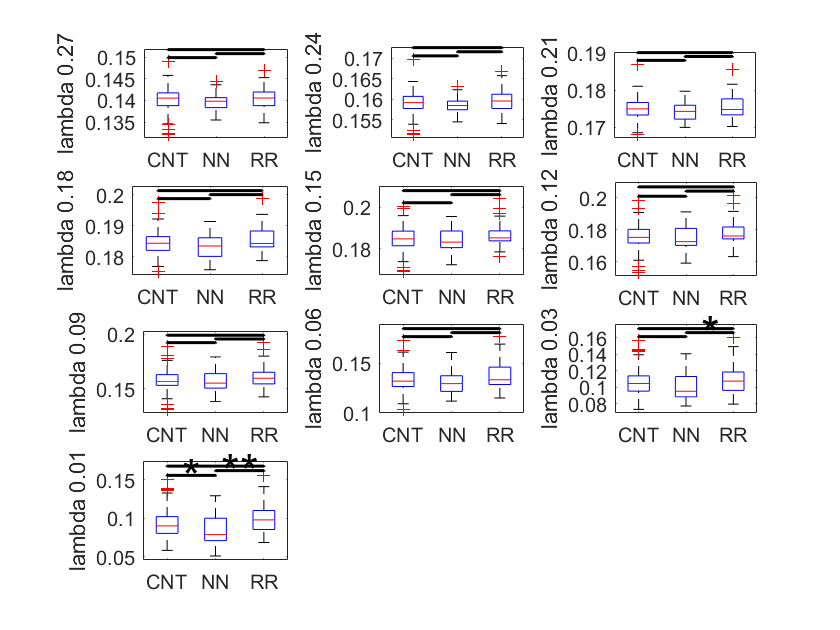


Supplementary Figure 1: Model-free turbulence result comparison between healthy controls (CNT) and non-responder/responder patient’s dichotomization: Turbulence across all scales ((from λ=0.01 to 0.27 in 0.03 steps). In the figure, P-values were assessed using the Wilcoxon rank-sum permutation test, and False discovery rate corrected: *P < 0.05, **P < 0.01 and ***P < 0.001.


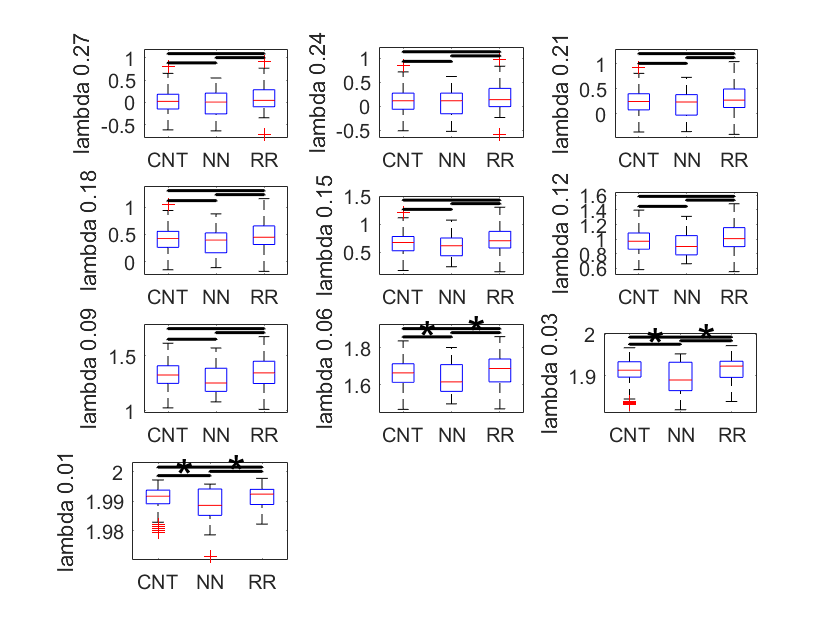


Supplementary Figure 1: Model-free information transfer result comparison between healthy controls (CNT) and non-responder/responder patient’s dichotomization: Turbulence across all scales ((from λ=0.01 to 0.27 in 0.03 steps). In the figure, P-values were assessed using the Wilcoxon rank-sum permutation test, and False discovery rate corrected: *P < 0.05, **P < 0.01 and ***P < 0.001.


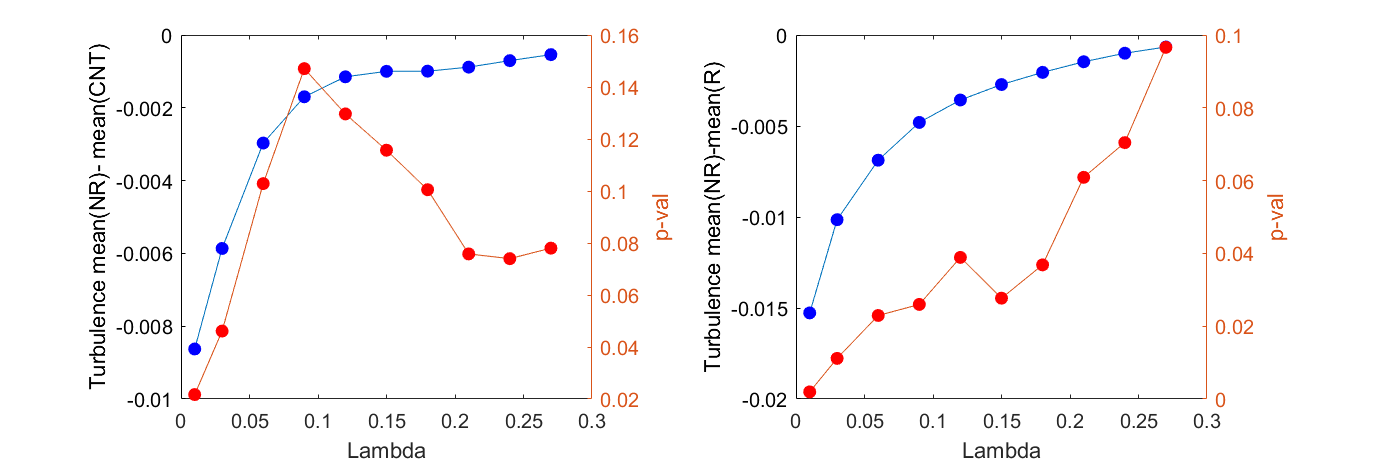


Supplementary Figure 3: difference between the mean of the turbulence between non-responder vs healthy controls as a function of the scale (lambda) (left). The difference of the mean non-responder and responder turbulence (right) as a function of the scale. In both cases the p-value of a Wilcoxon rank-sum permutation test (1000 repetitions) are also displayed as a function of the scale.
